# *In situ* studies of protein aggregation kinetics with multiparametric and superresolution imaging

**DOI:** 10.1186/1750-1326-8-S1-O15

**Published:** 2013-09-13

**Authors:** Gabriele Kaminski Schierle, Claire Michel, Eric Rees, Dorothea Pinotsi, Clemens Kaminski

**Affiliations:** 1 University of Cambridge, Cambridge, UK

## 

Misfolding and aggregation of peptides and proteins is a characteristic of many neurodegenerative disorders, including Parkinson’s (PD) and Alzheimer’s disease (AD). Their common feature is that normally unstructured and soluble proteins misfold and aggregate into insoluble amyloid fibrils, which make up the deposits in the brains of patients suffering from these devastating illnesses. A key requirement to gain insight into molecular mechanisms of disease and to progress in the search for therapeutic intervention is a capability to image the aggregation process and structure of ensuing fibrils *in situ*.

We have recently reported that amyloid proteins that are associated with protein misfolding diseases, including PD, AD and various other types of amyloidosis develop an intrinsic fluorescence in the visible range [1,2]. The discovery of intrinsic amyloid fluorescence has enabled the process of amyloid formation from disease-relevant polypeptides to be monitored in a label-free manner and with high specificity [2,3]. I will show here how specific and sensitive *in vivo* probes of amyloid structures can be developed using external fluorophores covalently attached to the amyloid backbone. Such external fluorophores can participate in Förster resonance energy transfer (FRET) with intrinsic energy states of amyloid structures if present, providing a readout in the form of a reduced fluorescence lifetime of the external fluorophores. I will provide an overview on the application of all-optical techniques to inform on the aggregation state of α-synuclein (relevant to PD), amyloid β and Tau (relevant to AD) *in vitro*, in live cells and model organisms. Multiparametric microscopy permits us to follow the kinetics of amyloid oligomerisation *in vivo* and correlate the appearance of aggregates with phenotypes of disease [1]. Using direct stochastic optical reconstruction microscopy, dSTORM, we are able to probe, in cells, the morphology of the ensuing aggregates with a resolution better than 20 nm [4]. We are able to distinguish different types of structures, including oligomeric assemblies and mature fibrils, and observe a number of morphological differences between the species formed *in vitro* and *in vivo*, which may be significant in the context of disease.
